# Attitudes towards depression and its treatment among white, hispanic, and multiracial adults

**DOI:** 10.1186/s40359-024-01804-8

**Published:** 2024-08-14

**Authors:** Leilani Feliciano, Kristi Erdal, Gro Mjeldheim Sandal

**Affiliations:** 1 Department of Psychology, University of Colorado, Colorado Springs, CO USA; 2 Department of Psychology, Colorado College, 14 East Cache la Poudre Street, Colorado Springs, CO 80903 USA; 3 Department of Psychosocial Science, University of Bergen, Bergen, Norway

**Keywords:** Depression, Attitudes, Culture, Treatment, Coping strategies, Multiracial adults

## Abstract

**Background:**

Depression is present in all societies and affects members of all racial and ethnic groups. However, attitudes about depression differ across groups and have been shown to impact help-seeking behaviors, preferences for treatments, and compliance with treatments.

**Methods:**

Taking a cross-cultural approach, this project used a case vignette of depression to examine race/ethnic group differences in attitudes about depression and its treatment among young adults in the U.S.

**Results:**

Data analyses revealed significant racial/ethnic group differences in attitudes as well as the treatments/strategies participants reported they would use. Gender x race/ethnicity interactions revealed that White and Multiracial/ethnic men were more likely to believe the vignette character should find a partner to help with symptoms, while White and Multiracial/ethnic women did not endorse those strategies. Hispanic men and women did not show a gender difference in that strategy, but gender differences were observed in other strategies. In a rare comparison, majority-minority Multiracial/ethnic participants (i.e., White selected as one of their races/ethnicities) rated identified helpers and treatments similarly to White participants and significantly higher than multiple-minority Multiracial participants (i.e., White not selected as one of their races/ethnicities).

**Conclusions:**

Findings supported previous research that indicates different U.S. racial/ethnic group ideas of depression and its treatment are potentially linked with cultural values, and we suggest that investigating these more fine-grained group differences can help to inform treating professionals as well as public health messages.

## Introduction

Depression is one of the most commonly experienced mental health concerns in adulthood. 5% of adults globally experience a depressive disorder, and depressive disorders are associated with substantial disability [1]. Depressive disorders are present in all societies and affect members of all cultural and ethnic groups [2–4]. In the U.S. in 2020, the prevalence of depressive disorder was approximately 8%, with some studies reporting higher rates of symptomatology in certain populations (e.g., low income, primary care patients) and after global disasters or events such as the COVID-19 pandemic [5, 6]. However, the way in which these disorders are understood and expressed (e.g., verbally as emotional or somatic complaints; behaviorally) seems to differ from society to society, and between ethnic groups [7, 8]. Similarly, the actions taken (e.g., recognition that symptoms may benefit from treatment, subsequent treatment seeking, engagement in treatment regimens, etc.) also differ by group [9, 10]. Taking a cross-cultural approach, this project examined U.S. racial/ethnic group differences in ideas about coping with and treating depression. A particular focus was to highlight race/ethnic group and gender differences in endorsement of coping and treatment strategies.

### Cross-cultural models for understanding depression

Scientific knowledge is “culturally situated” [11], implying that knowledge must always be interpreted in relation to the society and the context in which it is created. The cultural meaning of “being depressed” or “having depression” varies widely with the patient’s language, family system, and differentiation of emotional terminology. Prevalent in Western societies is the biomedical model in which psychiatric disorders are explained as being rooted in anatomy, heredity, and disease processes, and mental health problems are categorized in predefined diagnostic categories [12]. Alternative conceptualizations of mental health conditions endorsed by psychology, counseling, and social work fields include a biopsychosocial view of health in which the biological aspects of psychological disorders are still accounted for, but the social context in which behavior and physical functioning occurs is also taken into consideration [13]. However, even in a biopsychosocial model, the categorical conceptualization of mental disorders is still dominant.

Conversely, a ‘‘situational’’ model in which depression is embedded in the social context seems to be more prevalent in Eastern societies (e.g., Korea) and in minority communities in the U.S. and other Western countries (e.g., Ecuador) [14]. Social context can be thought of as the experiences linked to social categories such as age cohort, gender, ethnicity, as well as, coping styles and access to resources. Attributing depression to situational factors such as adverse life experiences and living conditions often contributes to seeing depressive symptoms as a normal reaction to adversities [15–17]. For example, South Asian women living in the U.S. describe the etiology of their depression as being due to more social factors (e.g., marital conflict) than biological factors. Similarly, older Korean immigrants describe depressive symptoms in terms of loneliness and family discord. Likewise, communities in other western countries like Ecuador maintain the notion of pena, which is a condition which has symptoms similar to depression (e.g., anhedonia, disturbed sleep, difficulties concentrating) but is considered more situational in nature, as it occurs after a “personal loss” [10, 18]. Research has also found that communities’ explanatory models of illness are associated with a wide range of clinically relevant variables, such as help-seeking behavior [19, 20], preferred treatment [21, 22], treatment compliance [23], and the therapeutic relationship [24]. Causal explanations are strongly linked with the kind of help perceived as most adequate and useful. In general, adherence to the situational model of mental illness has been associated with preference for seeking help and support from family and informal networks and more negative attitudes towards professional treatment [14].

Furthermore, among African American populations, mental illness, and depression in particular, is considered to be related to personal weakness, a lack of spirituality, or personal responsibility [25]. So, too, in Latinx/Hispanic populations, depression and distress are seen not only to be related to losses and problems in the family and social world, but also, that support from others is seen as the remedy for that distress or as at least necessary in approving chosen remedies [26–29]. This mismatch between these groups and the majority culture has important implications, as the likelihood of help-seeking in these minoritized groups may be much lower if the help sought and the help available are incongruent. It may be of no surprise then, that in countries like the U.S., differences between majority and minority groups in accessing and accepting mental health care differ greatly [28–30].

### Factors that impact help-seeking

In addition to the explanatory models of illness, other cultural variables may impact help-seeking, including level of religiosity, stigma, and emotional expression. Religiosity and stigma may both lead to less professional help seeking for mental illness, yet religiosity may result in better coping with stressful events, as people rely on their beliefs to derive meaning and hope, and provide a supportive community for those with depression [31, 32]. Chatters and colleagues [33] reported that African Americans drew strength from religion in difficult times and that this impacts the likelihood of seeking professional help for those who need it, as these individuals may be more likely to turn to their church group or faith, rather than an external agency (e.g., primary care provider, mental health clinician). While higher religiosity in Latinx women has been found to be associated with less willingness to seek counseling, it was mediated through beliefs in the spiritual etiology of mental illness [34]. Likewise, Latinx men have also reported faith in God as an important strategy to remedy depression, rarely seeing depression as a ‘chemical imbalance’ [26]. In Latinx populations, stigma has been found to be high for seeking help outside the family/community, as that may be seen as both a spiritual failure and a failure of that family/community [34], increasing negative emotions such as guilt and discouragement for feeling they have not lived up to their religious standards [31]. These internalized negative stereotypes of help-seeking and mental illness contribute to self-stigma when mental illness symptoms appear [35]. Cadaret and Speight [36] found that as levels of self-stigma increased in African-American males, negative attitudes towards help seeking for mental illness also increased.

Emotional expression of mental illness also differs both cross culturally and situationally. Thus, symptoms of depression may be expressed differently in different cultures and can vary by social context (i.e., age, gender) [37]. Younger adults may express many classic depressotypic symptoms, including sadness, lack of interest in activities (apathy), sleep disturbances, appetite changes, feelings of worthlessness, guilt, and thoughts of death or dying (suicidal ideation). Older adults with depression, however, may present with fewer emotional (e.g., sadness or low mood) or cognitive complaints (e.g., worthlessness or guilt) and endorse more somatic complaints (e.g., fatigue or muscle aches) [38–40], leading to a misattribution of symptoms as chronic illness and less treatment seeking for depression in this population.

Different ethnic/cultural groups may also report different symptoms of depression. For example, Sethi et al. [41] found that the theme of guilt was not commonly seen amongst Indian people with depression. Sweetland et al.’s [42] meta-analysis concluded that the concept of “depression” did not have a direct equivalent in sub-Saharan Africa, with distress often manifesting as somatic and behavioral, rather than cognitive symptoms. Idioms of distress, such as ataque de nervios in Puerto Ricans and other Latinx populations, have been described as having loss of control as the main symptom, in addition to such symptoms as fatigue, anxiety, loss of appetite, suicidality, etc [27]. Paykel [43] stated that a lack of awareness of other cultures’ languages and metaphors to describe depression has resulted in an under-detection and therefore underestimation of the prevalence of depression in these settings. These disparities may hamper the recognition of a depressive state in intercultural patient-health personal encounters which are common in ethnically diverse societies such as the U.S.

Recent research has examined Multiracial groups’ experiences with depression, as this group is the most rapidly growing demographic in the U.S [44]. By definition, “multiracial” encompasses all individuals who self-identify as two or more races, such as Black, White, Asian, etc., while “multiethnic” refers to individuals who self-identify as two or more ethnicities, such as Hispanic or Middle Eastern, and both terms can be defined differently from study to study [45]. However, as heterogeneous as these groups are, when compared to those who identify as monoracial (e.g., Black, White), Multiracial students were more likely to endorse symptoms of depression [46–49]. Depression in this group is thought to be related to racial discrimination, which can come in various forms; from experiencing discrimination from family members to micro-aggressions of the community about “fitting in” and “passing,” based on skin tone [44]. While being more likely to meet criteria for a mental health condition, Multiracial college students were also the most likely to endorse “deal with issues on my own,” rather than seek professional or even informal help for mental illness [48].

Likewise, the gender difference in emotional expression of certain mental health conditions is important to examine. Women, in general, tend to seek mental health services more often (i.e., in the US, 44.9% among females and 34.2% among males) and are twice as likely to be diagnosed with a depressive disorder than men [50]. These patterns have also been noted in Hispanic populations [51], with more women endorsing depression than men. The reasons for these gender differences are unclear. There is some discussion in the literature that the gender discrepancy in prevalence rates of depression may be related to gender socialization processes with women being socialized to recognize and talk about their emotions more so than men, as well as differences in the behavioral expression of depression (e.g., sadness in women vs. irritability or anger in men) [52]. Regardless of the exact mechanism, gender may impact expression of emotion and thus depressive symptoms.

To sum, different race/ethnic/cultural groups may conceptualize, think about, and have different attitudes towards mental health conditions such as depression, which may impact the type of coping strategies and treatment sought for depressive symptoms. Depression is associated with substantial disability, creating a significant burden on patients and their relatives, and to the society as a whole. Thus, to enhance the effectiveness of mental health services, it is critical for us to understand peoples’ ideas about treatment of and coping with this disorder. The way in which depression is understood seems to differ between groups. Studies suggest that considering such differences is essential for building mental health services that meet the needs of the population as a whole, as these beliefs and attitudes may impact health behaviors including recognition that something is wrong, acknowledging the need for help and/or treatment, help-seeking, accepting a diagnosis, and the likelihood of recovery. The present project aimed to gain more knowledge about transcultural variations in ideas about depression, coping strategies and treatment. Specifically, we examined and compared expectations and beliefs about how to handle depression held by adults in the U.S. from different racial/ethnic groups. Our main research question was: What are the racial/ethnic differences in preferred coping strategies and treatment of depression? And are they impacted by gender?

## Materials & method

### Participants

Participants were recruited as part of a larger international research project on mental health issues conducted by the Society and Workplace Diversity Group, Faculty of Psychology, at the University of Bergen, Norway. Participants in the current subsample included 642 students at a U.S. western public university, participating in a research project for extra credit. The sample identified as 72.9% female, 26.5% male, and 0.6% other, and ranged from 19 to 55 years old (*M* = 23.75, *mode* = 19, *SD* = 5.42). Participants self-identified their race/ethnicity and were able to select multiple categories with 80.7% selecting White, 22.7% selecting Hispanic, Latino, or Spanish, 7.3% selecting African American or Black, 5.9% selecting Asian, and 4.5% selecting American Indian or Alaska Native as one of their categories. Multiple racial/ethnic categories were endorsed by 18.6% of participants.

Mutually exclusive comparison groups were comprised of White (*n* = 421), Hispanic (*n* = 74), and Multiracial/ethnic (*n* = 119) participants. The Multiracial/ethnic group consisted of those who included White in their multiple races/ethnicities (majority-minority Multiracial; *n* = 98), and those who did not include White in their multiple races/ethnicities (multiple-minority Multiracial; *n* = 21).^1^

### Materials

**Vignette.** This online study asked participants to read a vignette of a gender-matched individual displaying depressive symptoms in line with the International Classification of Diseases – 10 [53]. Please see Appendix for the vignette. This vignette was previously used by Erdal et al. [54], with only the vignette characters’ names modified. Participants were then asked a series of follow-up questions about what they perceived as appropriate for the vignette character in dealing with the situation. Participants were asked to report how likely from 1 (very unlikely) to 6 (very likely) they would be to seek help from a list of 21 different types of people/helpers (e.g., parent, colleague, doctor) if they were feeling like the vignette character. They then indicated their agreement from 1 (strongly disagree) to 6 (strongly agree) with 32 different treatments and coping strategies for the vignette character (e.g., medication, rest, do nothing) [17]. 

### Procedure

Advertisement of the study was open in the Student Portal for 7 months during the academic year and students enrolled in the study electronically. Once enrolled, they completed an informed consent, the demographic form, and were asked to read and answer questions about the vignette as noted above. Once they completed the study, they were given a code to exchange for extra credit. This protocol was approved by the university Institutional Review Board.

## Results

### Dependent measures

#### People/identified helpers

The 21 questions about the types of people from whom participants might seek help were subjected to principal components factor analysis with a varimax rotation, resulting in a five-factor solution (with Eigenvalues over 1.0). The five factors were: Friends/Family (e.g., friends, parents, family; α = 0.66), Mental Health Professionals (e.g., psychologist, doctor; α = 0.65), Partner (1-item factor), Non-Mental Health Professionals (e.g., colleague, internet, co-student; α = 0.79), and Non-traditional (e.g., elder, alternative medicine, ethnic leader, traditional healer; α = 0.87).

#### Treatments/strategies

The 32 questions about treatments and strategies participants thought the vignette character should use were subjected to principal component factor analysis with a varimax rotation, resulting in an eight-factor solution (with Eigenvalues over 1.0). The eight factors were: Medication (1-item factor), Reflection (e.g., rest, reflection, have courage; α = 0.68), Externalizing (e.g., blame others, use alcohol, stay home; α = 0.73), Religious (e.g., pray to God, be prayed for; α = 0.91), Somatic (e.g., herbs, exercise, diet; α = 0.67), Partnering (e.g., find a partner, marry; α = 0.73), Time Off (e.g., vacation, leisure, stop and think; α = 0.67), and Overreacting (e.g., shame, no reason to be sad, do nothing; α = 0.82).

Factors identified from these analyses became the dependent variables of this study.

### Race/ethnicity and gender

Factorial ANCOVAs assessed the main effects and interactions of the three race/ethnicity groups (White, Hispanic, Multiracial/ethnic) and two genders (male, female) on each of the five People/Identified Helpers variables and the eight Treatment/Strategies variables. Age was covaried in the analyses through the use of a logarithmic transformation of age (ln age), as age was positively skewed. Means and standard deviations for the People/Identified Helpers and Treatment/Strategies variables are presented in Tables 1 and 2.

G*Power 3.1.9.7 was used to conduct a power analysis for ANOVA main effects and interactions. With α set at 0.05, power at 0.80, and estimating anticipated moderate effects (effect size *f* = 0.25), the total sample required was 158. To detect small-medium effects (effect size *f* = 0.15), the total sample required was 432. Due to multiple analyses, a Benjamini-Hochberg correction was run to limit false positives without reducing power as much as more conservative procedures. All analyses reported below at the *p* < .05 level are considered statistically significant.

**Table 1:** Means and standard deviations of the people/identified helpers variables

		M	(SD)	*n*
Non-Traditional	White	1.91	(0.90)	409
	Males	1.88	(0.94)	93
	Females	1.92	(0.88)	316
	Hispanic	1.81	(0.85)	69
	Males	2.02	(0.94)	21
	Females	1.73	(0.80)	48
	Multiracial/ethnic	2.03	(1.03)	114
	Males	1.98	(0.94)	40
	Females	2.06	(1.08)	74
Non-MHP	White	2.36	(0.91)	409
	Males	2.5	(0.94)	93
	Females	2.32	(0.90)	316
	Hispanic	2.23	(0.90)	69
	Males	2.22	(0.79)	21
	Females	2.23	(0.95)	48
	Multiracial/ethnic	2.4	(0.93)	114
	Males	2.43	(0.96)	40
	Females	2.39	(0.91)	74
MHP	White	3.55	(1.27)	409
	Males	3.54	(1.31)	93
	Females	3.55	(1.27)	316
	Hispanic	3.3	(1.41)	69
	Males	3.48	(1.43)	21
	Females	3.22	(1.40)	48
	Multiracial/ethnic	3.43	(1.35)	114
	Males	3.5	(1.41)	40
	Females	3.4	(1.32)	74
Friends/Family	White	4.15	(1.10)	409
	Males	4.03	(1.04)	93
	Females	4.18	(1.11)	316
	Hispanic	4.26	(1.20)	69
	Males	4.19	(1.02)	21
	Females	4.29	(1.27)	48
	Multiracial/ethnic	4.01	(1.09)	114
	Males	4.08	(1.00)	40
	Females	3.97	(1.14)	74
Partner	White	4.68	(1.36)	409
	Males	4.58	(1.35)	93
	Females	4.71	(1.36)	316
	Hispanic	4.94	(1.30)	69
	Males	4.71	(1.27)	21
	Females	5.04	(1.32)	48
	Multiracial/ethnic	4.73	(1.38)	114
	Males	5	(1.24)	40
	Females	4.58	(1.43)	74

**Table 2:** Means and standard deviations of the treatment/strategies variables

		M	(SD)	*n*
Overreacting*	White	2.05	(0.79)	404
	Males	2.42	(0.89)	91
	Females	1.94	(0.72)	313
	Hispanic	2.11	(0.88)	69
	Males	2.07	(0.81)	21
	Females	2.12	(0.91)	48
	Multiracial/ethnic	2.07	(0.78)	114
	Males	2.27	(0.80)	40
	Females	1.96	(0.76)	74
Somatic*	White	4.38	(0.69)	404
	Males	4.11	(0.68)	91
	Females	4.46	(0.68)	313
	Hispanic	4.35	(0.79)	69
	Males	4.22	(0.66)	21
	Females	4.41	(0.83)	48
	Multiracial/ethnic	4.24	(0.77)	114
	Males	4.14	(0.60)	40
	Females	4.29	(0.85)	74
Religious	White	3.21	(1.43)	404
	Males	3.37	(1.56)	91
	Females	3.16	(1.38)	313
	Hispanic	3.28	(1.52)	69
	Males	2.84	(1.47)	21
	Females	3.47	(1.51)	48
	Multiracial/ethnic	3.15	(1.53)	114
	Males	3.18	(1.48)	40
	Females	3.14	(1.57)	74
Externalizing*	White	1.79	(0.74)	404
	Males	2.08	(0.86)	91
	Females	1.71	(0.68)	313
	Hispanic	1.72	(0.71)	69
	Males	1.94	(0.64)	21
	Females	1.62	(0.73)	48
	Multiracial/ethnic	1.91	(0.81)	114
	Males	2.15	(0.74)	40
	Females	1.79	(0.82)	74
Time Off	White	3.68	(0.82)	404
	Males	3.81	(0.75)	91
	Females	3.64	(0.84)	313
	Hispanic	3.95	(0.93)	69
	Males	3.77	(0.79)	21
	Females	4.03	(0.98)	48
	Multiracial/ethnic	3.78	(0.86)	114
	Males	3.78	(0.82)	40
	Females	3.77	(0.89)	74
Partnering**, *	White	2.5	(1.06)	404
	Males	3.02	(1.16)	91
	Females	2.35	(0.97)	313
	Hispanic	2.54	(1.05)	69
	Males	2.55	(1.17)	21
	Females	2.54	(1.01)	48
	Multiracial/ethnic	2.78	(1.18)	114
	Males	3.38	(1.13)	40
	Females	2.46	(1.08)	74
Reflection	White	4.47	(0.80)	404
	Males	4.59	(0.76)	91
	Females	4.44	(0.81)	313
	Hispanic	4.78	(0.92)	69
	Males	4.56	(0.88)	21
	Females	4.88	(0.93)	48
	Multiracial/ethnic	4.5	(0.88)	114
	Males	4.68	(0.63)	40
	Females	4.4	(0.98)	74
Medication	White	3.24	(1.25)	404
	Males	3.29	(1.27)	91
	Females	3.22	(1.24)	313
	Hispanic	3.03	(1.40)	69
	Males	3	(1.28)	21
	Females	3.06	(1.47)	48
	Multiracial/ethnic	3.09	(1.28)	114
	Males	3.28	(1.20)	40
	Females	2.99	(1.32)	74

Note: * significant main effect of Gender

** significant Race/Ethnicity x Gender interaction

#### People/identified helpers

**Fig. 1:**
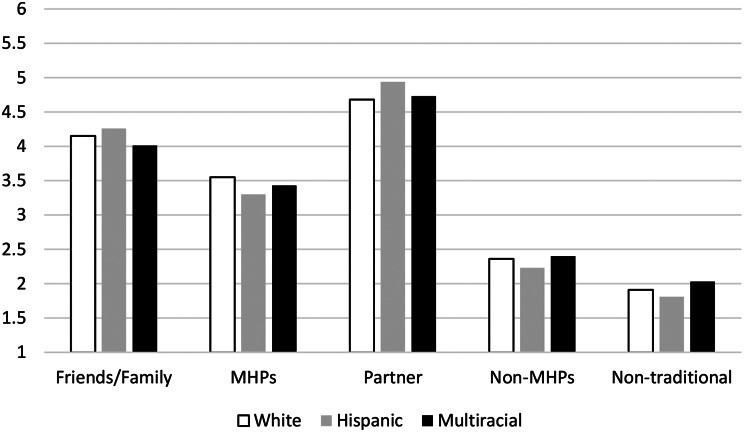
Racial/ethnic groups’ likelihood of seeking help from different people/identified helpers. *Note*. Scale of help-seeking likelihood (1–6). MHP = Mental Health Professionals

There were no significant main effects for Race/Ethnicity or Gender and no significant Race/Ethnicity x Gender interactions (all *p*s > 0.05) for any of the People/Identified Helpers, suggesting similarities in the way these university students approached help-seeking across races/ethnicities and genders. Please see Fig. 1.

#### Treatments/strategies

**Fig. 2:**
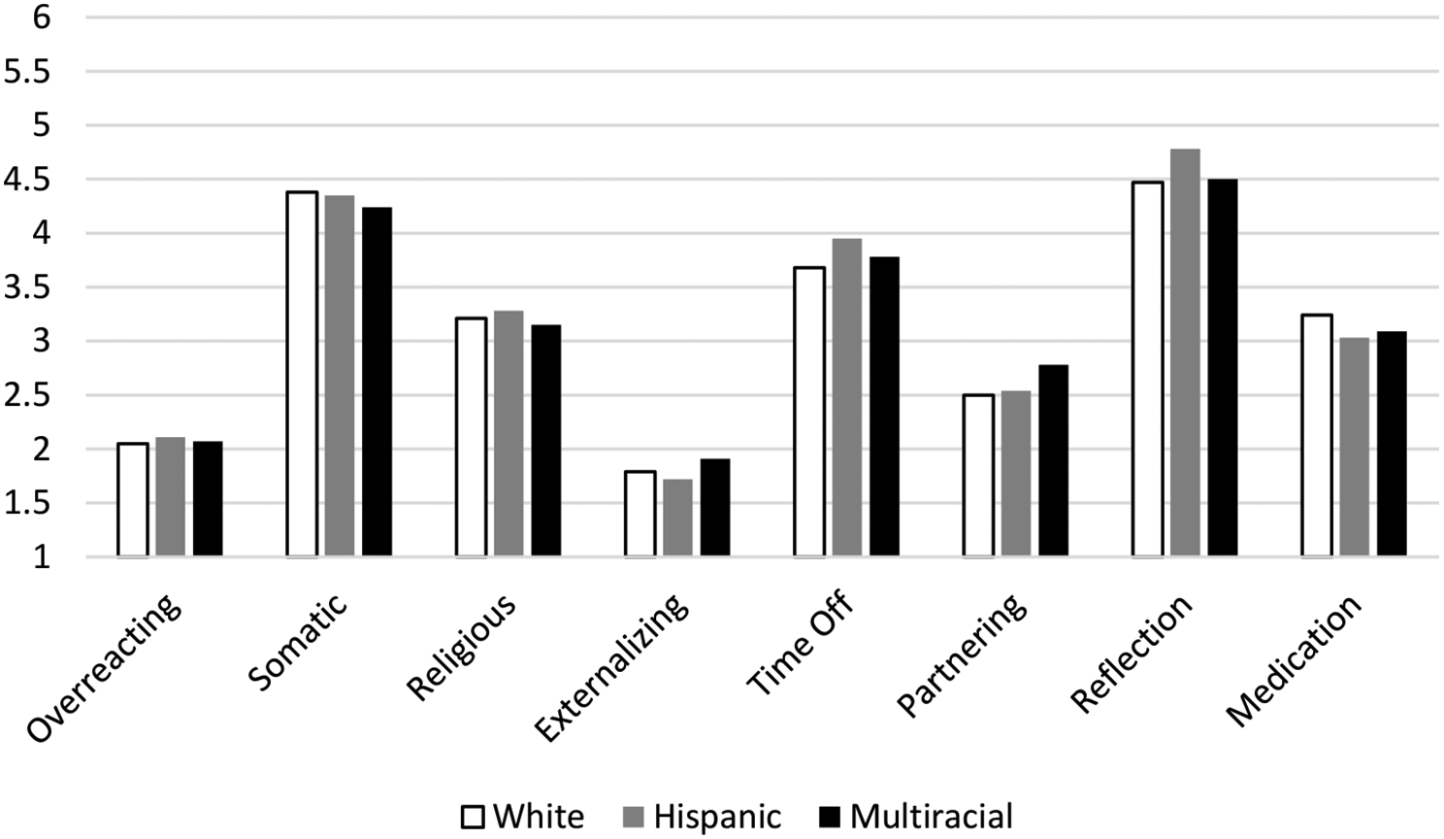
Racial/ethnic groups’ likelihood of using different treatment/strategies. *Note*. Scale of likelihood to use Treatment/Strategies (1–6)

The likelihood of different race/ethnic groups’ use of different treatments/strategies are presented in Fig. 2. There were no significant main effects of Race/Ethnicity or Gender, and no Race/Ethnicity x Gender interactions for Religious strategies, Time Off strategies, Reflection, or Medication (all *p*s > 0.05).

There was a significant main effect of Gender for Somatic treatments, *F* (1, 580) = 7.94, *p* = .005, η_p_^2^ = 0.014, such that females were more likely to endorse treatments such as diet and exercise. There were no main effects of Race/Ethnicity, *F* (2, 580) = 0.51, *p* = .60, η_p_^2^ = 0.002, nor a Race/Ethnicity x Gender interaction, *F* (2, 580) = 0.80, *p* = .45, η_p_^2^ = 0.003 for Somatic treatments. Age was not a significant covariate, *p* = .992.

There was a significant main effect of Gender on the use of Externalizing strategies, *F* (1, 580) = 18.06, *p* < .001, η_p_^2^ = 0.03, showing that males were more likely to endorse strategies such as alcohol use and blaming others. There were no main effects of Race/Ethnicity, *F* (2, 580) = 1.01, *p* = .36, η_p_^2^ = 0.003, nor a Race/Ethnicity x Gender interaction, *F* (2, 580) = 0.007, *p* = .99, η_p_^2^ = 0.000, for Externalizing strategies. Age was a significant covariate, *p* = .004.

There was a significant main effect of Gender on Overreacting, *F* (1, 580) = 7.82, *p* = .005, η_p_^2^ = 0.01, showing that males were more likely to believe that the vignette character should feel shame and do nothing. There was no main effect of Race/Ethnicity, *F* (2, 580) = 0.59, *p* = .56, η_p_^2^ = 0.002. There was no Race/Ethnicity x Gender interaction, *F* (2,580) = 2.49, *p* = .084, η_p_^2^ = 0.008. Age was a significant covariate, *p* = .021.

**Fig. 3:**
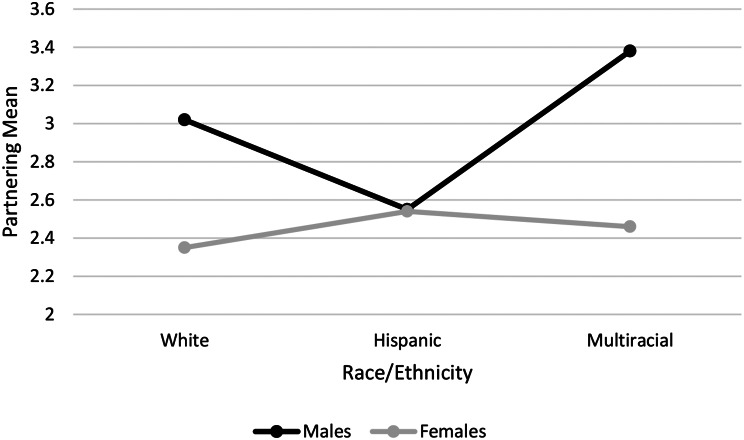
Race/ethnicity X gender interaction on beliefs the vignette character should find a partner as a treatment strategy. *Note.* Scale of Treatment/Strategy endorsement (1–6)

There was a significant Race/Ethnicity x Gender interaction for Partnering, *F* (2, 580) = 3.50, *p* = .031, η_p_^2^ = 0.012. Please see Fig. 3, which shows that White and Multiracial/ethnic males were more likely to believe the vignette character would profit from finding a Partner, White and Multi-racial/ethnic females were less likely to believe the vignette character would profit from finding a Partner, while Hispanic males and females were similar and reported a moderate endorsement of Partnering. There was also a significant main effect of Gender, *F* (1, 580) = 21.12, *p* < .001, η_p_^2^ = 0.035, showing that males were more likely to endorse Partnering as a treatment strategy. There was no main effect of Race/Ethnicity, *F* (2, 580) = 2.37, *p* = .094, η_p_^2^ = 0.008. Age was a significant covariate, *p* < .001.

### Multiracial/ethnic comparisons

In several analyses above, the Multiracial/ethnic group responded to questions about treatment strategies most similarly to the White group. In assessing the constituents of the Multiracial/ethnic group, they consisted of those who endorsed White as one of their races/ethnicities (majority-minority Multiracial/ethnic; *n* = 98) and those who did not endorse White as one of their races/ethnicities (multiple minority Multiracial/ethnic; *n* = 21). To explore any differences between these subgroups, one of which was of low *n*, Bayesian statistics were conducted for independent samples [55]. Bayesian statistics allow the comparison of the predictive ability of the null hypothesis (there were no differences between the two Multiracial/ethnic groups) to the alternative hypothesis (there was a difference between the two Multiracial/ethnic groups) to predict the observed data. Default priors were used in the analyses [55, 56]. BF_01_ refers to the Bayes factor in favor of the null hypothesis.

#### People/identified helpers

**Fig. 4:**
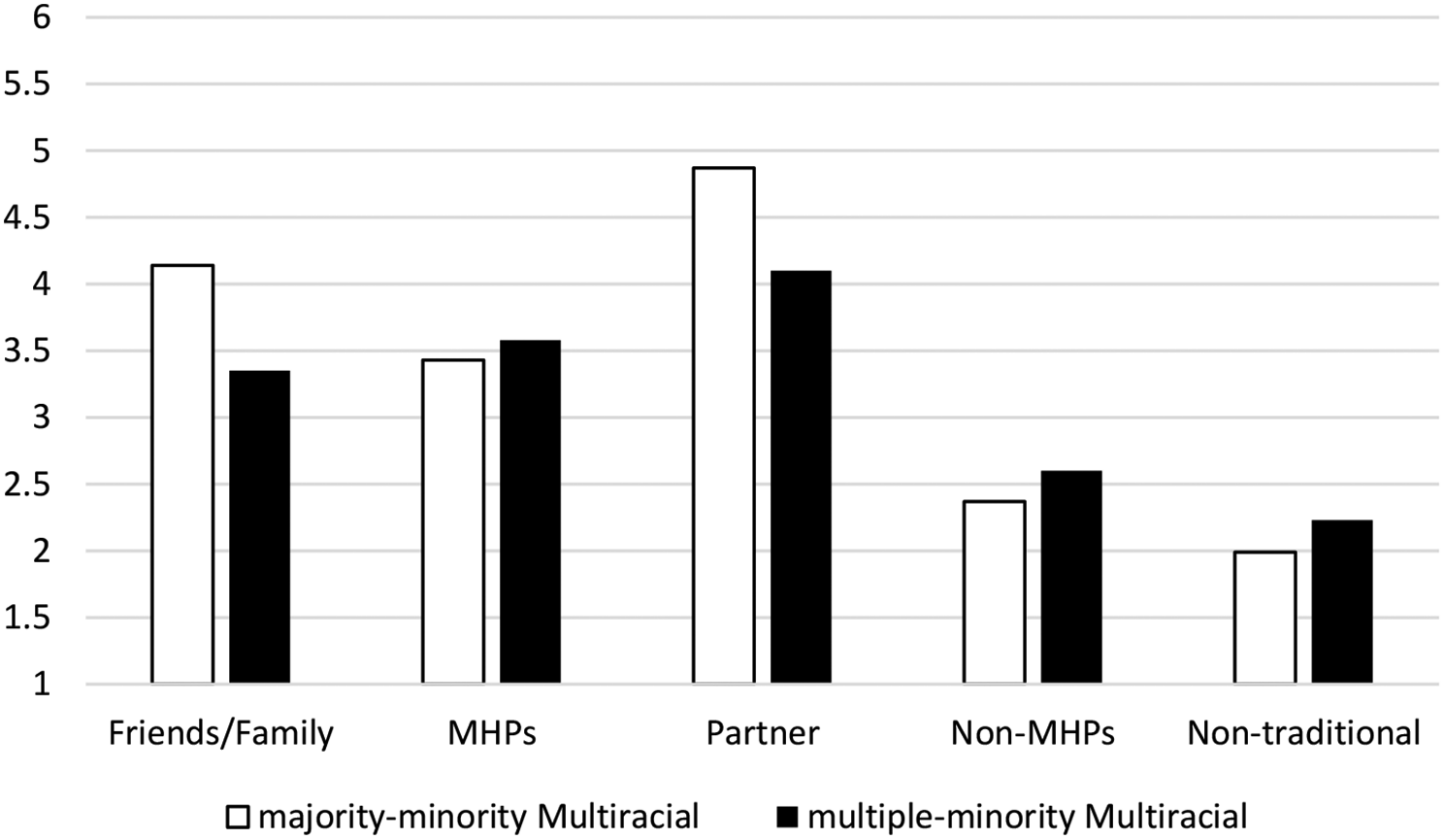
The people/identified helpers endorsed by the two groups of multiracial participants. *Note.* Scale of help-seeking likelihood (1–6)

Please see Table 3; Fig. 4.

For seeking help from Friends/Family, the BF_01_ suggested that the data were more probable under the alternative hypothesis than the null hypothesis. Given the size of the BF_01_, there was no support for the null hypothesis and strong support for the alternative hypothesis. Indeed, the data were 14.08 times (1/0.071) more likely under the alternative hypothesis than the null hypothesis. Those in the majority-minority Multiracial/ethnic group reported intent to seek help from Friends/Family more than those in the multiple-minority Multiracial/ethnic group.

For seeking help from Partners, the BF_01_ suggested that the data were inconclusive and equally likely under the null or alternative hypotheses.

For seeking help from Non-traditional practitioners, Non-Mental Health Professionals, and Mental Health Professionals, the BF_01_s suggested that the data were more probable under the null hypothesis. Given the sizes of the BF_01_s, there was moderate support for the null hypotheses. Indeed, the data were 3– 4x more likely under the null hypothesis than the alternative hypothesis for Non-traditional practitioners, Non-Mental Health Professionals, and Mental Health Professionals, suggesting no group differences.

**Table 3:** Means, standard deviations, and Bayesian statistics for the people/identified helper comparisons between the two multiracial/ethnic groups

Variables		Mean (SD)	Bayes	95% Credible Interval
	Multiracial/Ethnic		Factor	for the Mean
	Groups		(BF_01_)	Difference
Friends/Family			0.071	[-1.429, -0.1797]
	Majority-minority	4.15 (1.0)		
	Multiple-minority	3.35 (1.26)		
MHPs			4.95	[-0.5009, 0.7878]
	Majority-minority	3.43 (1.39)		
	Multiple-minority	3.58 (1.24)		
Partner			0.472	[-1.641, 0.0942]
	Majority-minority	4.87 (1.25)		
	Multiple-minority	4.10 (1.77)		
Non-MHPs			3.39	[-0.2845, 0.7417]
	Majority-minority	2.37 (0.91)		
	Multiple-minority	2.60 (1.03)		
Non-traditional			3.52	[-0.3630, 0.8508]
	Majority-minority	1.99 (0.98)		
	Multiple-minority	2.23 (1.23)		

Note: BF_01_ represents the evidence in support of the null hypothesis

BF_01_ < 0.10 = strong evidence for the alternative hypothesis (i.e., group differences)

BF_01_ 0.10 − 0.33 = moderate evidence for the alternative hypothesis (i.e., group differences)

BF_01_ 0.33–3.0 = equally supportive of the null and alternative hypothesis

BF_01_ 3–10 = moderate evidence for the null hypothesis

BF_01_ 10 + = strong evidence for the null hypothesis

#### Treatments/strategies

**Fig. 5:**
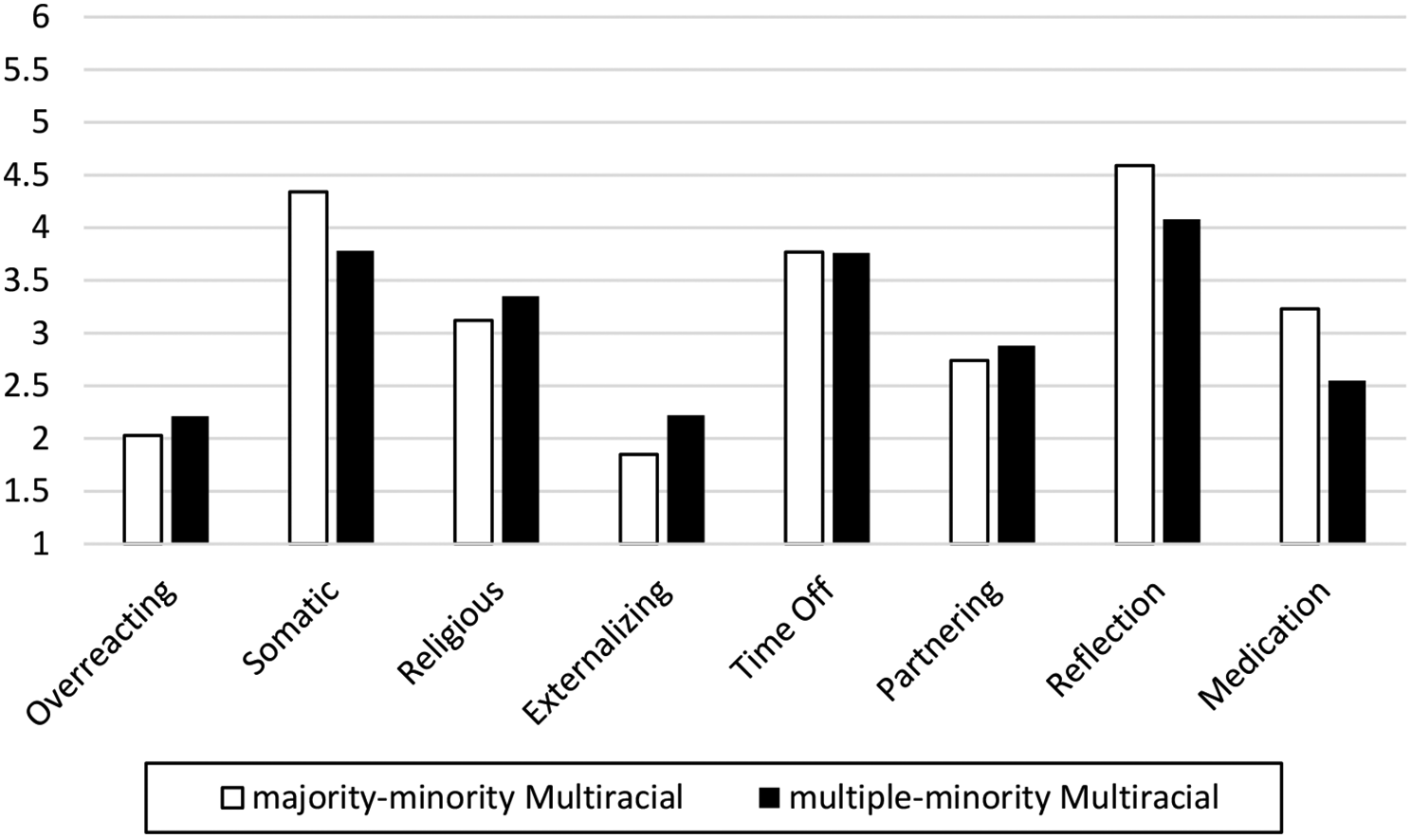
The treatments/strategies endorsed by the two groups of multiracial participants. *Note.* Scale of Treatment/Strategy endorsement (1–6)

Please see Table 4; Fig. 5.

For endorsement of Somatic treatments, the BF_01_ suggested that the data were more probable under the alternative hypothesis than the null hypothesis. Given the size of the BF_01_, there was no support for the null hypothesis and strong support for the alternative hypothesis. Indeed, the data are 11.11 times (1/0.09) more likely under the alternative hypothesis than the null hypothesis. Those in the majority-minority Multiracial/ethnic group reported endorsement of Somatic treatments more than those in the multiple-minority Multiracial/ethnic group.

For endorsement of Externalizing, Reflection, and Medication, the BF_01_s suggested that the data were inconclusive and equally likely under the null or alternative hypotheses.

For endorsement of Overreacting, Religion, Time Off, and Partnering, the BF_01_s suggested that the data were more probable under the null hypothesis. Given the sizes of the BF_01_s, there was moderate support for the null hypotheses. Indeed, the data were 3–5x more likely under the null hypothesis than the alternative hypothesis for Overreacting, Religion, Time Off, and Partnering, suggesting no group differences.

**Table 4:** Means, standard deviations, and Bayesian statistics for the treatments/strategies comparisons between the two multiracial/ethnic groups

Variables		Mean (SD)	Bayes	95% Credible Interval
	Groups		Factor	for the Mean
			(BF_01_)	Difference
Overreacting			3.55	[-0.3257, 0.6942]
	Majority-minority	2.03 (0.72)		
	Multiple-minority	2.21 (1.04)		
Somatic			0.09	[-1.0922, -0.0173]
	Majority-minority	4.34 (0.64)		
	Multiple-minority	3.78 (1.11)		
Religious			4.53	[-0.6974, 1.1588]
	Majority-minority	3.12 (1.45)		
	Multiple-minority	3.35 (1.88)		
Externalizing			1.08	[-0.1668, 0.9058]
	Majority-minority	1.85 (0.72)		
	Multiple-minority	2.22 (1.10)		
Time Off			5.37	[-0.5675, 0.5754]
	Majority-minority	3.77 (0.79)		
	Multiple-minority	3.78 (1.17)		
Partnering			4.89	[-0.6739, 0.9397]
	Majority-minority	2.74 (1.06)		
	Multiple-minority	2.88 (1.66)		
Reflection			0.405	[-1.0884, 0.0761]
	Majority-minority	4.59 (0.77)		
	Multiple-minority	4.08 (1.20)		
Medication			0.658	[-1.3498, -0.0134]
	Majority-minority	3.23 (1.28)		
	Multiple-minority	2.55 (1.32)		

Note: BF_01_ represents the evidence in support of the null hypothesis

BF_01_ < 0.10 = strong evidence for the alternative hypothesis (i.e., group differences)

BF_01_ 0.10 − 0.33 = moderate evidence for the alternative hypothesis (i.e., group differences)

BF_01_ 0.33–3.0 = equally supportive of the null and alternative hypothesis

BF_01_ 3–10 = moderate evidence for the null hypothesis

BF_01_ 10 + = strong evidence for the null hypothesis

## Discussion

The present project aimed to gain more knowledge about race/ethnic variations in the ideas about efficient coping and treatment of depression. Specifically, we examined and compared expectations and beliefs about depression treatments/strategies held by college aged adults in the U.S. from different racial/ethnic groups and genders. The data revealed several consistencies with previous research, such as gender’s effect on externalizing behaviors [57] and the use of somatic techniques for treatment [58], which cut across race/ethnicity. The data also revealed a gender effect on overreaction which suggest that masculine gender norms lead men to believe that they should be able to cope with depression without help (do nothing) and self-stigmatize (shame) if they cannot [48, 59, 60], decreasing both men’s informal and professional help-seeking [28, 59].

### People/identified helpers

Contrary to previous findings, we found no significant differences between racial/ethnic groups on the type of people that they would be willing to go to for help [32, 33]. Specifically, while we did not have an adequate sample size of people who identified exclusively as African-American to analyze in our study, our results for other minoritized groups did not support these findings (i.e., for our Hispanic nor Multiracial participants). One explanation for this discrepancy may be related to the level of religiosity. We did not explicitly assess for the level of religiosity subscribed to by our sample, and thus it is possible that we drew a sample of participants with lower levels of religiosity. Research conducted by Pew in 2017 [61] revealed that in general, less than half of college graduates reported that religion plays an important part of their lives. Similarly, data from the Cooperative Institutional Research Program (CIRP) Freshmen indicated that the number of students who identify as having no religious affiliation has increased from 10 to 31% over the last 30 years [62]. As Choi et al. [34] discussed, self-selection into and exposure to Eurocentric higher education may provide or support non-spiritual explanations for mental illness, potentiating this finding among college students.

### Treatments/strategies

The data revealed that, in Partnering, Hispanic men and women responded quite similarly to each other, in contrast to the gender differences of other races/ethnicities (see Fig. 3). Hispanic men and women appeared to drive that interaction by being similar to each other, while the White and Multiracial/ethnic men and women were quite different from each other.

In Fig. 3, a gender x race/ethnicity interaction was revealed, when Partnering as a coping strategy was endorsed the most by White and Multiracial/ethnic men, endorsed the least by White and Multiracial/ethnic women, and endorsed moderately by both Hispanic men and women. The gender effect of the White and Multiracial/ethnic samples is consistent with relationship research which has shown differential effects of males and females on the emotions and depression symptoms of their partners. That is, female partners’ emotional support has been shown to be positively related to positive emotions in their male partners, but male partners’ emotional support is not consistently related to females’ emotions and subsequent depressive symptoms [63], suggesting that partnering can be an effective strategy for men dealing with depression, but perhaps not as effective for women.

The Hispanic men and women, however, did not demonstrate that gender difference. This may be due to Latinx cultures, particularly Mexican, valuing the establishment and maintenance of strong interpersonal relationships across genders, and partnering being seen as an extension of that value [64]. This finding would need to be replicated, however, for confidence.

### Multiracial/ethnic comparisons

The opportunity to investigate differences between Multiracial/ethnic groups was largely unprecedented and revealed that those in the majority-minority Multiracial/ethnic group had different viewpoints about help-seeking and treatment approaches from those in the multiple-minority Multiracial/ethnic group. The experience of those in the majority-minority Multiracial/ethnic group was more similar to those participants who identified as White, in both the higher endorsement of People/Identified Helpers (Friends/Family) and Treatments/Strategies (Somatic).

Research on self-identity, involving skin tone as well as politics, may inform these findings. For students in the majority-minority Multiracial/ethnic group, it is likely that their overall phenotype is more similar to the White majority, possibly decreasing the number of racist and discriminatory events they receive [44, 65]. Indeed, Multiracial youth who appeared White were more likely to identify as White, when they had that option [65]. It has been proffered that in not needing to think about their ethnicity as often [47], this may give these students a more similar life experience and perspective on depression and its treatment to White populations than to other Multiracial/ethnic populations [65]. From a socio-political standpoint, Puerto Ricans have been found to utilize mental health services significantly more than Cubans and Mexicans in the U.S., suggesting their identity as U.S. citizens, in comparison to other Latinx populations, may impact their service utilization [30].

Other research has shown that Multiracial groups score most similarly to White groups on diagnoses and treatments, including reliance on friends and family [48], consistent with the present results. This may suggest, like in the current study, that these Multiracial samples are composed largely of participants who identify White as part of their racial/ethnic background. Further research into these findings is warranted as it seems clear that when the constituents of Multiracial/ethnic groups are examined more closely, those with multiple-minority identification have very different experiences with familial and community relationships as well as SES that may distinguish their opinions on help-seeking from those in a majority-minority group [44].

### Limitations

The primary limitations of this study relate to generalizability. The sample was recruited from a U.S. public university. While the age range was large, the majority of students were of typical college age. While age as a covariate only had mild impacts, several variables (Externalizing, Overreacting, Partnering) were affected by age and perhaps should be explored further. Younger adults, compared to midlife adults, express depressotypic symptoms (i.e., sadness, sleep disturbances, guilt) and may therefore consider treatments through this symptom lens. However, even here, age-affected responses may not generalize to those who are not in college or have never attended college, or those attending college in other regions.

This university is considered an ‘Emerging Hispanic Serving Institution (HSI)’ and has 68% of students on financial aid. However, White students remained overrepresented in our sample and it is clear that the privilege of a college education may change cultural values and experiences in ways which, in all likelihood, affected our data. Completing this research in the U.S., which holds a different history of race and a different health care system from other nations should also suggest caution in generalizing beyond the U.S. Despite this limitation in scope, this study does represent an initial foray into examining a diverse group of American adults and their corresponding views of depression and its treatment.

A limitation which could inform future work in this area is to solicit information about participants’ experience with depression or other mental illness. It is possible that participants who have experienced depression would respond differently to the vignette and to the questions about treatments. Likewise, participants who have engaged with one or more different types of treatments and whose satisfaction may vary with their experience with different treatment strategies (e.g., depressive symptoms were well-treated with medication or therapy vs. those who did not have good treatment experiences) may respond differently to the questions. There remains no standard way of assessing with confidence such self-reports (e.g., self-reported professional diagnosis, self-diagnosis) which also may be confounded by socio-economic status and availability to mental health resources. In cross-cultural research, this is made even more difficult by different terminology, resources, and support for such diagnoses in different cultures. However, attempts should be made to enumerate and discern the impact of previous experience with depression on these ideas.

## Conclusion

These results have implications for medical and mental health providers in that having a deeper understanding of how different race/ethnic/cultural groups view depression and its treatment may lead to greater understanding of how to approach conversations about treatment options. Treatment acceptability or getting patient “buy in” on how to approach treatment for depression may impact patient adherence with prescribed treatments and follow-through with provider recommendations. A conflict between cultural beliefs about depression and the biomedical or biopsychosocial conceptualization of depression may result in a lack of trust in the system and/or treatments offered, thereby negatively impacting adherence rates and outcomes.

## Data Availability

The datasets used and/or analyzed during the current study are available from the corresponding author on reasonable request.

## Appendix

Vignette.

John/Ann is a 27-year old waiter in a restaurant. In the last few weeks, s/he has been experiencing feelings of sadness every day. John/Ann’s sadness has been continuous and s/he cannot attribute it to any specific event or to the seasons. It is hard for him/her to go to work every day; s/he had enjoyed his/her co-workers and working at the restaurant, but now s/he cannot find any pleasure in this. In fact, John/Ann has little interest in most activities that s/he once enjoyed. John/Ann is not married and lives near his/her brother. Usually, they enjoy going out together and with friends. But now s/he does not enjoy this anymore. John/Ann feels very guilty about feeling so sad and feels that s/he has let down his/her brother and friends. S/He has tried different work habits and new hobbies to become motivated again, but s/he cannot concentrate on these tasks. Even his/her brother has now commented that John/Ann gets distracted too easily and cannot make decisions. Since these problems began, s/he has been sleeping poorly every day; s/he has trouble falling asleep and often wakes up during the night. A few nights ago, as s/he lay awake trying to fall asleep, John/Ann began to cry because s/he felt so helpless.

## Footnotes

1 This paper used terms and spellings consistent with Atkin et al.’s [45] suggestions.

## References

[1] World Health Organization. (2022). Depression. https://www.who.int/news-room/fact-sheets/detail/depression.

[2] Herrman H, Kieling C, McGorry P, Horton R, Sargent J, Patel V. Reducing the global burden of depression: a Lancet–World Psychiatric Association Commission. Lancet. 2019;393(10189):e42–3. 10.1016/S0140-6736(18)32408-5. 30482607 10.1016/S0140-6736(18)32408-5

[3] Kessler RC, Angermeyer M, Anthony JC, Graaf R, Demyttenaere K, Gasquet I, Girolamo G, Gluzman S, Gureje O, Haro J, Kawakami N, Karam A, Levinson D, Medina Mora M, Oakley Browne M, Posada-Villa J, Stein D, Adley Tsang C, Aguilar-Gaxiola S, Jordi A, Lee S, Heeeringa S, Pennell B, Berglund P, Gruber M, Petukhova M, Chatterji S, Ustün B. Lifetime prevalence and age-of-onset distributions of mental disorders in. Volume 6. the World Health Organization’s World Mental Health Survey Initiative. World Psychiatry; 2007. pp. 168–76. PMC2174588 18188442

[4] World Health Organization. Depression and other Common Mental disorders: Global Health estimates. Geneva: World Health Organization; 2017.

[5] Ettman CK, Abdalla SM, Cohen GH, Sampson L, Vivier PM, Galea S. Prevalence of depression symptoms in US adults before and during the COVID-19 pandemic. JAMA Netw Open. 2020;3(9):e2019686. 10.1001/jamanetworkopen.2020.19686. 32876685 10.1001/jamanetworkopen.2020.19686 PMC7489837

[6] Feliciano L, Johanson KA, Okun ML, Walden A. Impacts of the coronavirus pandemic on the emotional and physical health of older adults compared with younger cohorts. Clin Gerontologist. 2022;45(1):45–57. 10.1080/07317115.2021.1966561. 10.1080/07317115.2021.1966561 34463221

[7] Ahmed K, Bhugra D. Diagnosis and management of depression across cultures. Psychiatry. 2006;5(11):417–9. 10.1053/j.mppsy.2006.08.010. 10.1053/j.mppsy.2006.08.010

[8] Haroz EE, Ritchey M, Bass JK, Kohrt BA, Augustinavicius J, Michalopoulos L, Burkey MD, Bolotn P. How is depression experienced around the world? A systematic review of qualitative literature. Soc Sci Med. 2017;183:151–62. 10.1016/j.socscimed.2016.12.030. 28069271 10.1016/j.socscimed.2016.12.030 PMC5488686

[9] Gopalkrishnan N. Cultural diversity and mental health: considerations for policy and practice. Front Public Health. 2018;6:1–7. 10.3389/fpubh.2018.00179. 29971226 10.3389/fpubh.2018.00179 PMC6018386

[10] Karasz A. Cultural differences in conceptual models of depression. Soc Sci Med. 2005;60(7):1625–35. 10.1016/j.socscimed.2004.08.011. 15652693 10.1016/j.socscimed.2004.08.011

[11] Haraway D. Situated knowledges: the science question in feminism and the privilege of partial perspective. Feminist Stud. 1988;14(3):575–99. 10.2307/3178066

[12] Deacon BJ. The biomedical model of mental disorder: a critical analysis of its validity, utility, and effects on psychotherapy research. Clin Psychol Rev. 2013;33(7):846–61. 10.1016/j.cpr.2012.09.007. 23664634 10.1016/j.cpr.2012.09.007

[13] Renn BN, Feliciano L. Biopsychosocial theoretical framework. In: Wenzel AE, editor. The SAGE encyclopedia of abnormal and clinical psychology. Thousand Oaks, CA: SAGE Publications, Inc.; 2017. pp. 497–9.

[14] Karasz A. The development of valid subtypes for depression in primary care settings: a preliminary study using an explanatory model approach. J Nerv Mental Disease. 2008;196(4):289–96. 10.1097/NMD.0b013e31816a496e PMC2774710 18414123

[15] Aarethun V, Sandal G, Guribye E, Markova V, Bye H. Explanatory models and help-seeking for symptoms of PTSD and depression among Syrian refugees. Soc Sci Med. 2021;277. 10.1016/j.socscimed.2021.113889. 10.1016/j.socscimed.2021.113889 33838449

[16] Brea D, Sandal G, Guribye E, Markova V. Explanatory models of post-traumatic stress disorder (PTSD) and depression among Afghan refugees in Norway. BMC Psychol. 2022;10(1). 10.1186/s40359-021-00709-0. 10.1186/s40359-021-00709-0 PMC8728976 34983663

[17] Markova V, Sandal G. Lay explanatory models of depression and preferred coping strategies among Somali refugees in Norway. A mixed-method study. Front Psychol. 2016. 10.3389/fpsyg.2016.01435. 7. 27713719 10.3389/fpsyg.2016.01435 PMC5031692

[18] Chentsova-Dutton YE, Tsia JL. Understanding depression across cultures. In: Gotlib IH, Hammen CL, editors. Handbook of depression. 2nd ed. New York: NY. The Guilford; 2009.

[19] Compas B, Connor-Smith J, Saltzman H, Thomsen A, Wadsworth M. Coping with stress during childhood and adolescence: problems, progress, and potential in theory and research. Psychol Bull. 2001;127(1):87–127. 11271757 10.1037/0033-2909.127.1.87

[20] Guribye E, Sandal G, Oppedal B. Communal proactive coping strategies among Tamil refugees in Norway: a case study in a naturalistic setting. Int J Mental Health Syst. 2011. 10.1186/1752-4458-5-9. 5. 10.1186/1752-4458-5-9 PMC3096987 21521494

[21] Cabassa L, Hansen M, Palinkas L, Ell K. Azúcar Y nervios: explanatory models and treatment experiences of Hispanics with diabetes and depression. Soc Sci Med. 2008;66(12):2413–24. 18339466 10.1016/j.socscimed.2008.01.054 PMC2475593

[22] Iniguez E, Palinkas L. Varieties of health services utilization by underserved Mexican American women. J Health Care Poor Underserved. 2003;14(1):52–69. 12613068 10.1353/hpu.2010.0827

[23] deVries M. Culture, community and catastrophe: issues in understanding communities under difficult conditions. In: Hobfoll S, deVries M, editors. Extreme stress and communities: impact and intervention. New York, NY, US: Kluwer Academic/Plenum; 1995.

[24] Clauss-Ehlers C, Lopez Levi L. Violence and community, terms in conflict: an ecological approach to resilience. J Social Distress Homeless. 2002;11(4):265–78. 10.1023/A:1016804930977

[25] Black H, White T, Hannum S. The lived experience of depression in elderly African American women. Journals Gerontol. 2007;62(6):S392–8. 10.1093/geronb/62.6.S392 PMC4539960 18079427

[26] Cabassa L. Latino immigrant men’s perceptions of depression and attitudes toward help seeking. Hispanic J Behav Sci. 2007;29(4):492–509. 10.1177/0739986307307157

[27] Guarnaccia P, Rivera M, Franco F, Neighbors C. The experiences of ataques de nervios: towards an anthropology of emotions in Puerto Rico. Cult Med Psychiatry. 1996;20:343–67. 8899285 10.1007/BF00113824

[28] Selkirk M, Quayle E, Rothwell N. A systematic review of factors affecting migrant attitudes towards seeking psychological help. J Health Care Poor Underserved. 2014;25:94–127. 24509015 10.1353/hpu.2014.0026

[29] Villatoro A, Morales E, Mays V. Family culture in mental health help-seeking and utilization in a nationally representative sample of Latinos in the United States: the NLAAS. Am J Orthopsychiatry. 2014;84(4):353–63. 24999521 10.1037/h0099844 PMC4194077

[30] Keyes K, Martins S, Hatzenbuehler M, Blanco C, Bates, Hasin D. Mental health service utilization for psychiatric disorders among Latinos living in the United States: the role of ethnic subgroup, ethnic identity, and language/social preferences. Soc Psychiatry Psychiatr Epidemiol. 2012;47:383–94. 21290097 10.1007/s00127-010-0323-y PMC3756540

[31] Bonelli R, Dew RE, Koenig HG, Rosmarin DH, Vasegh S. (2012). Religious and spiritual factors in depression: Review and integration of the research. Depression Research and Treatment, 2012, 962860. 10.1155/2012/962860. 10.1155/2012/962860 PMC3426191 22928096

[32] Turner N, Hastings J, Neighbors H. Mental health care treatment seeking among African American and Caribbean Blacks: what is the role of religiosity/spirituality? Aging Mental Health. 2019;23(7):905–11. 29608328 10.1080/13607863.2018.1453484 PMC6168439

[33] Chatters L, Taylor R, Jackson J, Lincoln K. Religious coping among African Americans, Caribbean Blacks, and non-Hispanic Whites. J Community Psychol. 2008;36(3):371–86. 21048887 10.1002/jcop.20202 PMC2967036

[34] Choi N, Kim H, Gruber E. Mexican American women college students’ willingness to seek counseling: the role of religious cultural values, etiology beliefs, and stigma. J Couns Psychol. 2019;66(5):577–87. 31259575 10.1037/cou0000366

[35] Chan K, Mak W. Attentional bias associated with habitual self-stigma in people with mental illness. PLoS ONE. 2015;10(7). 10.1371/journal.pone.0125545. 10.1371/journal.pone.0125545 PMC4503620 26177536

[36] Cadaret MC, Speight L. An exploratory study of attitudes toward psychological help seeking among African American men. J Black Psychol. 2018;44(4):347–70. 10.1177/0095798418774655. 10.1177/0095798418774655

[37] Kirmayer L. Cultural variations in the clinical presentation of depression and anxiety: implications for diagnosis and treatment. J Clin Psychiatry. 2001;62(Suppl 13):22–8. 11434415

[38] Erdal K. Depressive symptom patterns in patients with Parkinson’s disease and other older adults. J Clin Psychol. 2001;57(12):1559–69. 11745597 10.1002/jclp.1118

[39] Hybels C, Blazer D, Pieper C, Landerman L, Steffens D. Profiles of depressive symptoms in older adults diagnosed with major depression: latent cluster analysis. Am J Geriatric Psychiatry. 2009;17(5):387–96. 10.1097/JGP.0b013e31819431ff PMC2718569 19390296

[40] Kim J, Shin I, Yoon J, Stewart R. Prevalence and correlates of late-life depression compared between urban and rural populations in Korea. Int J Geriatr Psychiatry. 2002;17(5):409–15. 11994928 10.1002/gps.622

[41] Sethi B, Nathawat S, Gupta S. Depression in India. J Soc Psychol. 1973;91(1):3–13. 4749504 10.1080/00224545.1973.9922640

[42] Sweetland A, Belkin G, Verdeli H. Measuring depression and anxiety in sub-Saharan Africa. Depress Anxiety. 2014;31:223–32. 23780834 10.1002/da.22142 PMC4109689

[43] Paykel E. Basic concepts of depression. Dialog Clin Neurosci. 2008;10(3):279–89. 10.31887/DCNS.2008.10.3/espaykel PMC3181879 18979941

[44] Marks L, Thurston I, Kamody R, Schaeffer-Smith M. The role of multiracial identity integration in the relation between racial discrimination and depression in multiracial young adults. Prof Psychology: Res Pract. 2020;51(4):317–24. 10.1037/pro0000315

[45] Atkin A, Christophe N, Stein G, Gabriel A, Lee R. Race terminology in the field of psychology: acknowledging the growing multiracial population in the US. Am Psychol. 2022;77(3):381–93. 35254853 10.1037/amp0000975 PMC9316411

[46] Chen J, Stevens C, Wong S, Liu C. Psychiatric symptoms and diagnoses among U.S. college students: a comparison by race and ethnicity. Psychiatric Serv. 2019;70(6):442–9. 10.1176/appi.ps.201800388 PMC6628693 30914001

[47] Fisher S, Reynolds J, Hsu W, Barnes J, Tyler K. Examining multiracial youth in context: ethnic identity development and mental health outcomes. J Youth Adolesc. 2014;43:1688–99. 25100614 10.1007/s10964-014-0163-2

[48] Lipson S, Kern A, Eisenberg D, Breland-Noble A. Mental health disparities among college students of color. J Adolesc Health. 2018;63:348–56. 30237000 10.1016/j.jadohealth.2018.04.014

[49] National Institute of Mental Health. (2023). Major depression. Retrieved from https://www.nimh.nih.gov/health/statistics/major-depression.

[50] Substance Abuse and Mental Health Services Administration, Center for Behavioral Health Statistics and Quality. (November 19, 2015). The NSDUH Report: Revised Estimates of Mental Illness from the National Survey on Drug Use and Health. Rockville, MD. 27656737

[51] National Institute of Mental Health. (2008). Prevalence of serious mental illness among U.S. adults by age, sex, and race. Retrieved from http://www.nimh.nih.gov/statistics/.

[52] Oliffe JL, Phillips MJ. Men, depression and masculinities: a review and recommendations. J Men’s Health. 2008;5(3):194–202.

[53] World Health Organization (WHO). The ICD-10 classification of mental and behavioural disorders. World Health Organization; 2007.

[54] Erdal K, Singh N, Tardif A. Attitudes about depression and its treatment among mental health professionals, lay persons and immigrants and refugees in Norway. J Affect Disord. 2011;133(3):481–8. 21620476 10.1016/j.jad.2011.04.038

[55] Krypotos A, Blanken T, Arnaudova I, Matzke D, Beckers T. A primer on Bayesian analysis for experimental psychopathologists. J Experimental Psychopathol. 2017;8(2):140–57. 10.5127/jep.057316 PMC5524172 28748068

[56] Tendeiro J, Kiers H, Hoekstra R, Wong T, Morey R. Diagnosing the misuse of the Bayes Factor in applied research. Adv Methods Practices Psychol Sci. 2024;7(1):1–19. 10.1177/25152459231213371

[57] Mehl-Madrona L, McFarlane P, Mainguy B. Epigenetics, gender, and sex in the diagnosis of depression. Curr Psychiatry Res Reviews. 2019;15:277–89.

[58] Salari-Moghaddam A, Keshteli A, Mousavi S, Afshar H, Esmaillzadeh A, Adibi P. Adherence to the MIND diet and prevalence of psychological disorders in adults. J Affect Disord. 2019;256:96–102. 31170621 10.1016/j.jad.2019.05.056

[59] Cole B, Ingram P. Where do I turn? Gender role conflict, self-stigma, and college men’s help-seeking for depression. Psychol Men Masculinities. 2020;21(3):441–52. 10.1037/men0000245

[60] Latalova K, Kamaradova D, Prasko J. Perspectives on perceived stigma and self-stigma in adult male patients with depression. Neuropsychiatr Dis Treat. 2014;10:1399–405. 25114531 10.2147/NDT.S54081 PMC4122562

[61] Pew Research Group. (2017). In America, Does More Education Equal Less Religion? Downloaded from https://www.pewforum.org/2017/04/26/in-america-does-more-education-equal-less-religion/.

[62] Downey A. (2017). College freshman are less religious than ever. Sci Am Downloaded from https://blogs.scientificamerican.com/observations/college-freshmen-are-less-religious-than-ever/.

[63] Gruen R, Gwadz M, Marrobel D. Support, criticism, emotion and depressive symptoms: gender differences in the stress-depression relationship. J Social Personal Relationships. 1994;11:619–24. 10.1177/0265407594114009

[64] Soto JA, Levenson RW, Ebling R. Cultures of moderation and expression: emotional experience, behavior, and physiology in Chinese Americans and Mexican Americans. Emotion. 2005;5(2):154–65. 15982081 10.1037/1528-3542.5.2.154

[65] Herman M. Forced to choose: some determinants of racial identification in multiracial adolescents. Child Dev. 2004;75(3):730–48. 15144483 10.1111/j.1467-8624.2004.00703.x

